# Genetic components associated with R2 and R4 powdery mildew resistance in hop

**DOI:** 10.1002/tpg2.70180

**Published:** 2026-02-11

**Authors:** Klara Hajdu, John Connell, Peter Darby, Michael Baldock, Andrew Armitage, Alastair Ainslie, Sarah Blackburn, Carol Wagstaff, Helen Cockerton

**Affiliations:** ^1^ NIAB East Malling UK; ^2^ Wye Hops Ltd., China Farm Office Canterbury UK; ^3^ Department of Food and Nutritional Sciences University of Reading Berkshire UK; ^4^ Natural Resources Institute University of Greenwich Kent UK; ^5^ School of Natural Sciences University of Kent Canterbury UK

## Abstract

Epidemics of powdery mildew disease in hop (*Humulus lupulus* var. *lupulus*) lead to cone spoilage, and in severe cases, crop abandonment. In order to prevent disease‐associated yield losses, hop must be treated with an intensive fungicide management program. However, the chemical toolbox available is shrinking, and the horticultural industry is now increasing the uptake of sustainable biological disease control strategies, including genic‐based resistance, which can offer a low chemical input strategy. We investigate the genetic components associated with powdery mildew resistance in (1) a hop population that segregates for R2 resistance and (2) a diversity panel containing 736 individuals with differential resistance responses to the hop powdery mildew race‐structure. Both populations were phenotyped using the “Zenith” isolate (V1, V3, and Vb virulence) and genotyped to enable the association mapping of the biparental population and a genome‐wide association study analysis, respectively. We identified the same location on chromosome 6 associated with R2 resistance in both populations. However, an additional resistance allele was associated with R4 resistance. Notably, the most significant single nucleotide polymorphisms on chromosome 6 fall on either side of three *RPM1* disease resistance genes, which are prime candidates for downstream analysis. RPM1 mediates a localized cell death disease response reminiscent of the R2 phenotype. These results provide novel validated markers for use in international hop breeding programs. In doing so, we facilitate the pyramiding of disease resistance genes against multiple races of powdery mildew and reduce reliance upon chemical applications through providing a varietal control solution for this major hop pathogen.

## Core Ideas

- The authors examined the facilitation of marker‐assisted breeding to enable efficient genetic improvement in hop.
- This research looks at reducing grower reliance upon fungicides through generating varietal control solutions against hop powdery mildew.
- Successful pyramiding of powdery mildew resistance requires a deeper understanding of the molecular mechanisms controlling powdery mildew R genes.

## Plain Language Summary

Crop breeding offers an environmentally positive strategy to protect crop production from disease epidemics while lessening the impact of agricultural chemicals on natural populations and reducing pesticide residues. Here, we study hop plants, the flowers of which are a key ingredient required to make beer. We identify the specific pieces of DNA that, when selected by breeders, can be used to create new disease‐resistant hops. Currently, hop growers must spray their crops with chemicals every 10 days during the growing season to prevent the establishment of the pathogen that causes powdery mildew disease. Through harnessing the valuable genetic markers we have identified, crop breeders will be able to develop high‐quality hops that are immune to the disease.

**Abbreviations**
ACD6: accelerated cell death 6
ANOVA: analysis of variance
BLUE: best linear unbiassed estimate
DArT: Diversity Arrays Technology Pty
HR: hypersensitive response
KW: Kruskal–Wallis
GWAS: genome‐wide association study
NS: nonsignificant
MAS: marker‐assisted selection
NS: nonsignificant
PM: powdery mildew
QTL: quantitative trait loci
R gene: resistance gene
RPM1: resistance to *Pseudomonas maculicola* 1
SNP: single nucleotide polymorphism

## 1 INTRODUCTION

The female flowers or cones of hop (*Humulus lupulus* var. *lupulus*) are the key ingredient imparting bitterness and aromatic flavors to beer. When developing new hop cultivars, breeders must balance the needs of hop growers and brewers. Hop growers require disease‐resistant cultivars with superior agronomic performance, whereas brewers demand high flavor cultivars with good brewing potential (Darby, 2005a; Nesvadba, 2016). For the past century, hop breeders have primarily implemented classical, phenotyping‐based selection approaches to improve desirable plant characteristics such as resin content, yield, and resistance to diseases such as Verticillium wilt, downy mildew, and powdery mildew (McAdam et al., 2013). Embracing genetic informed breeding strategies can increase the efficiency of disease‐resistant cultivar generation.

Powdery mildew (PM) is the most significant airborne fungal disease impacting hop production. In order to protect against the establishment of the disease, UK hop growers must spray hop plants with fungicides every 10 days during the season. Without active control of the disease, PM infection leads to cone spoilage and up to a 20% reduction in yield as measured through alpha acid content (Gent et al., 2014). Disease‐resistant varieties offer a favorable alternative to chemical disease control strategies. In current UK hop breeding, the powdery mildew resistance status of new breeding lines is assessed phenotypically, via mass glasshouse infection assays. *Podosphaera macularis* (Walr.), the causal agent of hop powdery mildew, is an obligate biotroph meaning that inoculum must be maintained on living hop plants in order to undertake a PM infection assay. To put the costs of phenotyping into perspective, disease screening for powdery mildew requires the all‐year‐round maintenance of the pathogen on susceptible inoculator plants grown in light‐ and temperature‐controlled growth facilities with the transfer of *P. macularis* onto fresh hop leaf tissues every 7–14 days. This inoculum is used in glasshouse pathogenicity experiments to select resistant individuals from breeding populations (Darby, 2013).

Conversely, molecular marker‐assisted selection (MAS) enables individuals to be selected based upon DNA marker patterns that are associated with a disease‐resistant phenotype rather than through observing the phenotype directly. MAS may be a favorable alternative to glasshouse disease screening, especially where conducting low‐cost laboratory tests on young hop seedlings can significantly reduce the cost and time of phenotypic confirmation in breeding lines (Čerenak et al., 2019). However, for the successful implementation of MAS, we first require a comprehensive understanding of the genetic components conferring the trait of interest.

Hop powdery mildew resistance predominantly presents as a qualitative phenotype, controlled by discrete major genes that follow Mendelian segregation in seedling populations (Darby, 2001), and disease resistance breeding in hop has traditionally focused on the introgression of these “vertical” resistances that are controlled by single major resistance genes (R genes). There is a comprehensive system defining sources of hop powdery mildew resistance based on differential host–pathogen interactions. To date, a series of seven race‐specific resistance sources, namely R1, R2, R3, R4, R5, R6, and Rb (resistance genes protecting hops against corresponding races of hop powdery mildew), have been described, each of which provides qualitative resistance in hop against specific pathotypes of *P. macularis* (Mahaffee et al., 2009). Recent studies have indicated that some PM resistance variation may be quantitative, under the control of multiple small effect genes (Henning et al., 2024; Padgitt‐Cobb et al., 2020). Partial resistance which provides reduced disease severity, but not complete immunity, has been observed in hop (Darby et al., 1989; Twomey et al., 2015), with the cultivar Cascade considered one such example of partial resistance (Brooks & Horner, 1971; Gent et al., 2017).

Historically, different parental sources of resistance have been employed in each hop breeding program, depending upon the race of *P. macularis* present in the target growing region (Darby, 2001). For example, R2 resistance based on the cultivar Wye Target and R4 resistance derived from the cultivar Serebrianka are currently the main sources of powdery mildew resistance deployed in the United Kingdom and Europe, while up until recent years resistance provided by the R6 gene (originally identified in the cultivar Nugget) has been the most effective source of resistance against North American races of *P. macularis* (Block et al., 2021; Darby, 2013; Gent et al., 2017; Godwin et al., 1987; Wolfenbarger et al., 2016).

Once identified, monogenic resistance is relatively easy to breed into new cultivars compared to quantitative resistance; however, major gene resistance is prone to being overcome by highly virulent pathogen races. These races have evolved in response to the selection pressure experienced where plants containing a single source of resistance are planted across a large geographical area (Miedaner et al., 2020). One such example where hop resistance has been overcome by new pathogen races is the loss of the monogenic powdery mildew R6 resistance provided by the Nugget cultivar (Eriksen et al., 2023). Unfortunately, due to the evolution of highly virulent *P. macularis* strains, some of the PM R‐genes that have been deployed in hop cultivars have been overcome (Havill et al., 2023; Wolfenbarger et al., 2016). Consequently, US breeding programs are switching to utilizing R1 and R2 PM resistance in new hop cultivars, while breeding efforts in the United Kingdom and Europe are moving toward the use of R4 and R6 resistance sources (Wolfenbarger et al., 2016). In order to generate durable powdery mildew resistant hops, multiple resistance sources should be stacked into a single cultivar (Gent et al., 2018; Mundt, 2018). Successful pyramiding of powdery mildew resistance requires a deeper understanding of the molecular mechanisms controlling powdery mildew R genes.

The use of molecular markers to complement a traditional breeding strategy is becoming more achievable for hop breeding programs. In particular, MAS offers a strategy for rapid improvement in the durability of the qualitative disease resistances for powdery mildew through pyramiding of major R genes into a single cultivar, which would provide multi‐layered protection against different pathogen races (Merrick et al., 2021). To support the generation of resilient powdery mildew resistant hop cultivars, this study aimed to identify genetic markers to replace the labor‐intensive disease phenotyping assays currently employed in hop breeding programs.

**Core Ideas**

- The authors examined the facilitation of marker‐assisted breeding to enable efficient genetic improvement in hop.
- This research looks at reducing grower reliance upon fungicides through generating varietal control solutions against hop powdery mildew.
- Successful pyramiding of powdery mildew resistance requires a deeper understanding of the molecular mechanisms controlling powdery mildew R genes.

## 2 MATERIALS AND METHODS

### 2.1 Plant material

A biparental mapping population was developed from the female parent “Pilgrim” and male parent 316/01/10 (the male accession used in the mapping population) at the Wye Hops breeding program (China Farm, Canterbury, Kent; Hieronymus, 2012). The UK cultivar Pilgrim is resistant to major hop diseases and carries the R2 type powdery mildew resistance, while the male accession 316/1/10 has a susceptible phenotype. The diversity panel was composed of 736 genotypes from the Wye Hops germplasm collection including historic and named cultivars, public accessions, wild hop genotypes, genotypes derived from wild accessions, and important breeding lines. Plant material was treated with a standard pest and disease spray management program during propagation and routine growth.

### 2.2 Phenotypic data collection

For all plant material used in this study, powdery mildew resistance status was phenotyped using the methods described by Darby (2005b). The Pilgrim x 316/1/10 population was phenotyped in both 2016 and 2022. In 2016, powdery mildew resistance was recorded for 107 individuals at the seedling stage with a single seedling assessed per genotype. However, in 2022, 107 perennial clonally propagated genotypes were assessed in an incomplete randomized block design with one to five replicates per genotype, depending upon plant viability. Clonal propagation of parents and individuals for disease phenotyping assays was carried out at National Institute of Agricultural Botany (East Malling, UK) via soft shoot cuttings taken from established, 4‐year‐old field genotypes maintained in field plots at China Farm. The diversity panel was phenotyped across 38 experiments conducted between 1982 and 2022 (excluding 2016 and 2021) as individual seedlings alongside replicated standards. The hop cultivar Zenith was included in all experiments to confirm pathogen race specificity of the *P. macularis* ‘Zenith’ isolate which possessed V1, V3, and Vb virulence (hop powdery mildew races corresponding to resistance genes; Neve & Darby, 1983). The standard cultivar Wye Target carrying the resistance source R2 and the universal susceptible genotype Northern Brewer were also included in all experiments (Godwin et al., 1987). Additional hop PM differential standard cultivars carrying the resistance sources R3 (Challenger), R4 (Serebrianka), R5 (Early Choice), R6 (Nugget), R2Rsov (Sovereign), and Rq (Cascade; Darby, 2001; Gent et al., 2017; Neve, 1972, 1986; Neve & Darby, 1982; Salmon, 1948), and susceptible genotypes “Columbus” and “Yeoman” were included as clonally propagated controls in multiple experimental years (Table S1). Each of these genotypes exhibit a defined response to inoculation with the Zenith PM isolate (Figure S1).

**FIGURE 1:**
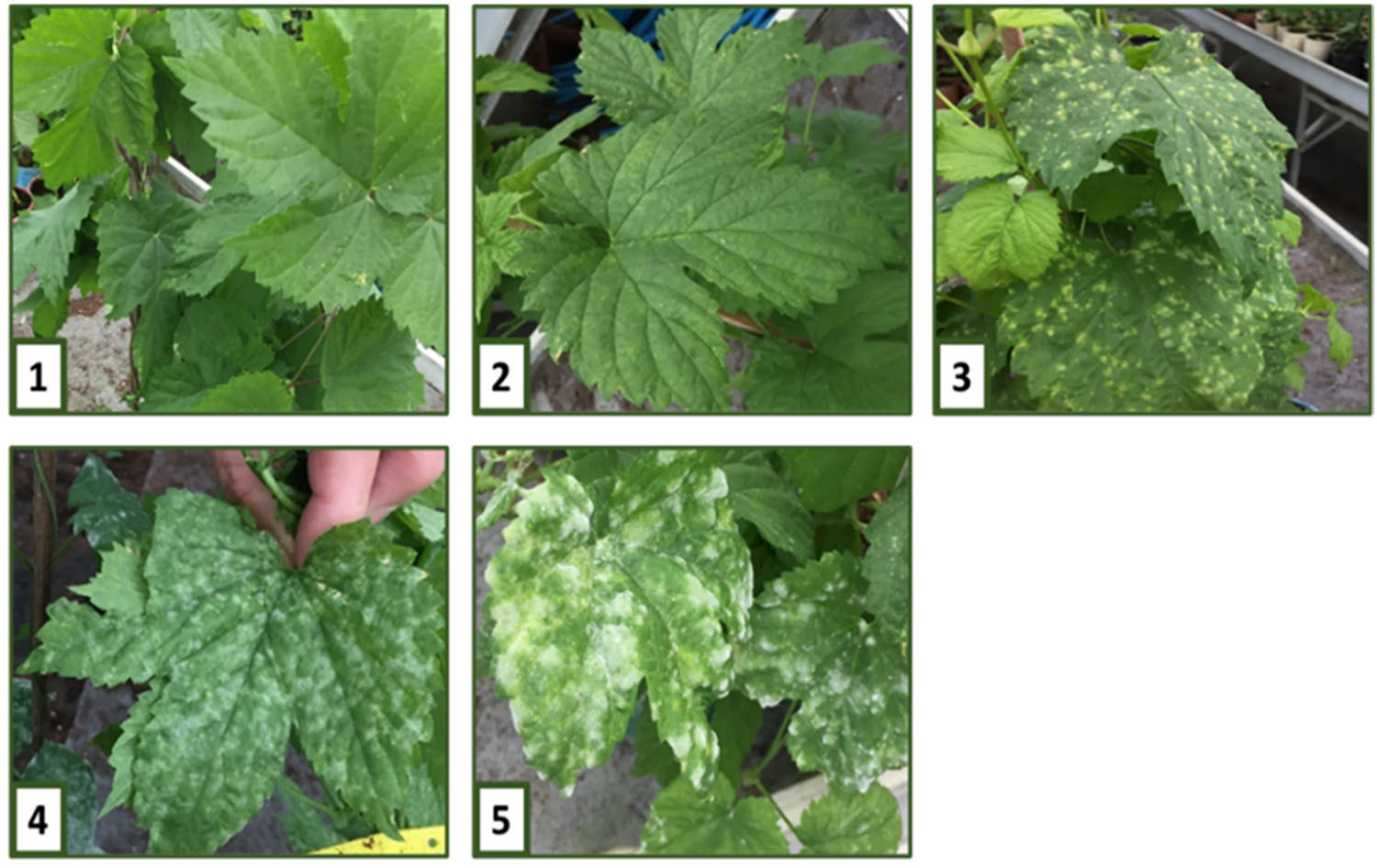
Hop powdery mildew disease severity scores. (1) “Resistant,” no infection; (2) “Poor resistant,” chlorosis may occur; (3) “Very poor resistant,” chlorosis, blistering, and aborted sporulation; (4) “Query susceptible,” ghost sporulation in glasshouse condition, weak resistance in field conditions; and (5) “Susceptible,” necrosis, chlorosis, merging lesions, and sporulation on petioles and under the leaves.

The PM scores of the individual genotypes were normalized based on the severity of infection observed in the R2 resistant (Wye Target, Sovereign, Pilgrim) and susceptible (Zenith, Northern Brewer) differential standard cultivars within each experiment. The actual PM scores were compared to the standard cultivars expected scores. The difference between expected and observed scores was used as a correction factor in each experiment. This normalization allowed the raw PM scores to be adjusted for all genotypes so that results were consistent and comparable across trials. Inoculum was bulked up in vivo, via a series of inoculum transfers onto the highly susceptible Northern Brewer genotype. Inoculated Northern Brewer plants were incubated in a temperature‐ and light‐controlled growth cabinet at 15°C on 12/12 h photoperiod. All experiments were conducted using the *P. macularis* Zenith isolate. The Zenith PM isolate has been shown to cause a discontinuous granular series of disease symptoms in families segregating for the R2 gene and complete immunity in R4, R5, and R6 segregating families (Darby, 2013). The classification of seedling reactions in glasshouse testing was ranked on a 1–5 ordinal scale based upon the severity of sporulation on individuals where 1 is considered fully resistant/immune, 2 as resistant, 3 as weak resistant/query resistant, 4 as partially susceptible, and 5 as fully susceptible phenotypes (Figure 1). Artificial PM inoculation assays in all experimental years were conducted in a glasshouse compartment by inoculating approximately 30‐cm tall potted plants. Powdery mildew inoculation was achieved by providing an even and dense covering of conidia shaken from Northern Brewer inoculator plants suspended 1.5 m above the experimental plants. Experimental plants were maintained at 20°C in a 14:10 h light:dark cycle for 16–20 days until the assessment of disease symptoms. Powdery mildew symptoms were recorded 15 days after inoculation as a single assessment with the plants that scored very poor resistant (3) or query susceptible (4) confirmed 20 days after inoculation.

### 2.3 Statistical analysis of phenotypic data

The observed segregation ratio of resistance provided by the R2 gene in the Pilgrim x 316/1/10 family was tested for goodness of fit against a 1:1 chi‐squared distribution with an alpha threshold of 0.05, with a nonsignificant (NS) *p*‐value indicating no deviation from the 1:1 ratio, computed using the chisq.test() function in the “stats” R package (R Core team, 2016). A two‐way analysis of variance (ANOVA) was performed to assess the effect of genotype, experiment year, and block on the powdery mildew disease phenotypes. A spatial P‐spline model was created based on row and plot positions of each plant within randomized blocks with genotype set as a random factor using the “SpATS” R package (Rodríguez‐Álvarez et al., 2018). The overall powdery mildew disease scores for each genotype were generated with a mixed linear model using the “lmer()” function of the “lme4” R package to combine single phenotype scores from 2016 and spatially autocorrelated best linear unbiased estimates from 2022, with genotype and year set as random effects (Bates et al., 2015). For the diversity panel, a linear mixed model was used to calculate disease resistance status for each hop line (assigned as a fixed variable), across replicate years (assigned as a random variable) with scores adjusted in reference to standard cultivars. Broad‐sense heritability (*H* ^2^) of the powdery mildew resistance trait was calculated from the variance components obtained from the mixed model analysis using the formula *H* ^2 ^= *V*(*g*)/*V*(*p*), where *V*(*g*) is the total genotypic variance and *V*(*p*) is the phenotypic variance.

### 2.4 Genotypic data generation

DNA was extracted from small unexpanded leaves (<1 cm in length) for the biparental population, using the NucleoSpin 96 Plant II DNA extraction kit (Macherey‐Nagel). DNA from four replicates of the genotypes Pilgrim, 316/1/16, and Cascade and single replicates of the 172 progenies were submitted to Diversity Arrays Technology Pty (DArT; Bruce, Australia) for genotyping by sequencing. Of the 172 progenies genotyped, a subset (107) was phenotyped due to the availability of plant material. Small unexpanded leaves for 739 genotypes that made up the diversity panel were freeze dried for 24 h, and 10–15 mg of sample was frozen in liquid nitrogen and milled for 3 min before submission to DArT for DNA extraction and genotyping by sequencing. Methylation sensitive restriction enzymes were used to produce reduced complexity sequencing libraries. The rare cutting *Pst*I and the frequently cutting *Mse*I restriction enzyme endonucleases were used as described in the protocol developed by Kilian et al. (2012). Restriction fragments were sequenced on an Illumina HiSeq 2000 platform, returning an average sequencing yield of 1.2 million reads per sample.

### 2.5 Single nucleotide polymorphism variant calling and functional annotation

Paired‐end Illumina WGS and DArT‐Seq raw reads were trimmed for adapters and low‐quality base calls using trimgalore version 0.6.10 with flags ‐q25 and –length 75 to remove low‐quality base calls and to specify a minimum read length of 75 bp (Krueger, 2015). Trimmed sequencing reads were aligned to the chromosome‐level Dovetail Cascade reference assembly (Padgitt‐Cobb et al., 2023) using BWA‐mem version 0.7.17‐r1188 with the addition of read group information (Li, 2013). Read alignments were quality‐filtered using SAMtools version 1.19.2 with flags ‐q20 ‐F256 to filter for a minimal mapping quality value of 20, leaving only primary alignments (Li et al., 2009). Variant sites were identified using BCFTools version 1.9 with the commands mpileup and called with the additional flags C50 to adjust mapping quality to the recommended value, and ‐a AD, DP to add allelic and read depth information to the output VCF file (Li & Barrett, 2011). A total of 10, 347 markers were used in the association mapping study with 14, 859 used in the genome‐wide association study (GWAS) analysis.

The resulting VCF file was filtered using VCFtools version 0.1.16 (Danecek et al., 2011). Variants with a quality score less than 20, INDEL variants and variants that were identified within 5 bp of an INDEL start or end position were removed. Furthermore, multiallelic sites, variants with a read depth less than 5, and sites with more than 20% missing data or alleles with a frequency <5% were removed (MAF threshold set to protect against false positive associations and prioritize association detection across common variants). The resulting variants were annotated with Ensemble VEP version 113 (McLaren et al., 2016) using the Cascade Dovetail genome reference and gene models downloaded from HopBase.org (https://hopbase.org/Downloads.php). Gene functional annotations were generated using InterProScan version 5.74‐105 (P. Jones et al., 2014).

### 2.6 Biparental association mapping and GWAS analysis for powdery mildew resistance

Significant associations between powdery mildew resistance phenotypes and DArT single nucleotide polymorphism (SNP) genotypes of the Pilgrim x 316/1/10 progeny were assessed using the Kruskal–Wallis (KW) non‐parametric ANOVA at *p *= 0.05 after error correction with false discovery rate method for multiple hypothesis testing using the stats R package (R Core Team, 2016). The association mapping of molecular markers linked to powdery mildew resistance was performed separately upon phenotyping data recorded in 2016 and 2022 and upon the combined data using genotypic best linear unbiassed estimates (BLUEs) calculated using a linear mixed model for PM resistance. Powdery mildew disease phenotype and significant marker genotype associations were assessed using a KW analysis with post hoc comparisons to estimate mean differences between genotype classes.

To assess the association between resistance phenotypes from the diversity panel and DArT SNP genotypes, a GWAS analysis was performed. The mixed‐model GWAS was conducted using GEMMA via a high‐performance computer cluster (Zhou & Stephens, 2012) with the minor allele frequency cutoff of 0.05 and population structure controlled through inclusion of the kinship matrix. The significance values were corrected using a Benjamini–Hochberg test to reduce false discovery. A two‐way ANOVA was used to assess the interactions between focal SNPs. Coefficient of determination (*R* ^2^) values were calculated to estimate the effect size of significant SNP markers indicating the proportion of variation in powdery mildew resistance phenotypes explained by the identified SNPs. Statistical associations between marker genotypes and powdery mildew resistance sources were calculated using chi‐squared and Fisher's exact tests.

### 2.7 Investigation of powdery mildew resistance related genes

Genomic positions of DArT sequence tags containing significant powdery mildew resistance associated markers were determined using the Cascade Dovetail reference. Gene models within the powdery mildew resistance window of 308.7–312.8 Mb on chromosome 6 (defined via biparental association analysis) and 1 Mb window around the main SNP identified via the GWAS were investigated, and putative disease resistance genes were identified based on associations with the Biological Processes Gene Ontology (GO) terms “defence response,” “plant type‐hypersensitive response,” “defence response to fungus,” “ethylene activated signalling pathway,” and “carbohydrate metabolic processes” and the assigned putative functions of “Cascade” genes based on similarity to known UniProt Embryophyta proteins and Pfam domains as annotated by Padgitt‐Cobb et al. (2023). Gene tables documenting variant calls, surrounding genes, and gene functional annotations were produced using a custom python script.

## 3 RESULTS

Phenotypic variation associated with R2 resistance illustrated a 1:1 segregation ratio between resistant (disease scores 1–3) and susceptible (disease score 4–5) progeny in the Pilgrim x 316/1/10 family in 2016 (*χ* ^2^_(1)_ **= 0.29; *p *= 0.59) and 2022 (*χ* ^2^_(1)_ **= 0.35; *p *= 0.56) PM assay. There was no difference in powdery mildew phenotype scores between experimental replicates, and the spatial analysis revealed no significant deviation between the fitted spatial trend and the original phenotype scores. A broad‐sense heritability (*H* ^2^) score of 0.99 and 0.94 was obtained from the spatial analysis and ANOVA of the 2022 experiment, respectively, indicating the high heritability of the R2 powdery mildew trait. Broad‐sense heritability for powdery mildew resistance was estimated for the 12 standard cultivars included across multiple diversity panel experiments (Figure S2). The heritability was high (*H* ^2^ = 0.94).

A single PM‐associated region spanning 3.4 Mb was identified upon chromosome 6 in the Pilgrim x 316/1/10 population. KW analysis identified 12 SNPs significantly associated with powdery mildew resistance across both assessment years and the combined analyses using the overall phenotypic BLUEs. All significant markers co‐localized on chromosome 6 of the physical map in a 3.4‐Mb region spanning 309.9–313.3 Mb. Ten of the 12 significant markers segregated in the PM‐resistant female parent; the remaining markers segregated in the male parent. Effect sizes of the significant SNP markers ranged from 13% to 55% with the focal SNP positioned at 311,711,438 bp contributing 55% of the explained phenotypic variation (Table 1).

**TABLE 1:** Significant markers associated with R2 powdery mildew resistance, identified through association mapping in the Pilgrim x 316/1/10 biparental hop population.

SNP No	Chromosome 6 position (bp)	Reference allele	Alternative allele	Pilgrim genotype	316/1/10 genotype	Segregation type	Distribution of phenotypes	*χ* ^2^ (*df* = 1)	Marker useful	KW *p*‐value	Marker effect size (*R* ^2^)
1	311711437	C	T	C/T	—	AB x –	1:1	0.48 (*p *= 0.49)	y	<0.001	0.55
2	311711455	A	G	C/T	—	AB x –	1:1	0.48 (*p *= 0.49)	y	<0.001	0.55
4	310959501	A	G	A/G	T/T	AB x AA	2:1	0.02 (*p *= 0.89)	y	<0.001	0.40
3	310959493	G	T	G/T	T/T	AB x AA	2:1	0.01 (*p *= 0.9)	y	<0.001	0.40
6	309901110	T	A	T/A	T/T	AB x AA	2:1	0.06 (*p *= 0.81)	y	<0.001	0.28
5	309901108	T	C	T/C	T/T	AB x AA	2:1	0.02 (*p *= 0.89)	y	<0.001	0.22
8	313337835	T	C	T/C	T/T	AB x AA	2:1	0.41 (*p *= 0.52)	y	<0.001	0.21
9	309901071	A	T	A/T	A/A	AB x AA	2:1	0.81 (*p *= 0.37)	y	<0.001	0.20
7	313337823	T	C	T/C	T/T	AB x AA	2:1	0.32 (*p *= 0.57)	y	<0.001	0.19
10	313337878	T	C	T/C	T/T	AB x AA	2:1	0.66 (*p *= 0.42)	y	<0.001	0.15
11	311767362	C	T	T/T	C/T	AA x AB	2:01	0.04 (*p *= 0.84)	n	0.006	0.13
12	311767384	A	G	A/A	A/G	AA x AB	2:1	0.12 (*p *= 0.73)	n	0.006	0.00

*Note*: The “Marker useful” column denotes whether the SNP is segregated in the resistant parent.

Abbreviation: KW, Kruskal–Wallis.

**FIGURE 2:**
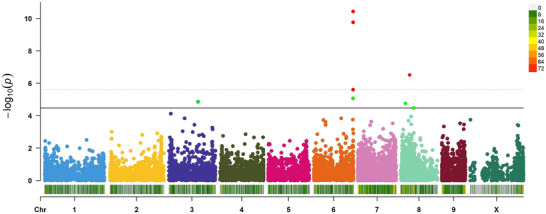
Manhattan plot denoting the significance of single nucleotide polymorphisms (SNPs) with powdery mildew resistance in the genome‐wide association study (GWAS) panel. Notations on the *x*‐axis (1–9) detail the chromosome number, and the sex chromosome is represented by X. The solid horizontal line represents the significance threshold of *p* = 0.05 after adjustment. The dotted horizontal line represents the significance threshold of *p *= 0.01 after adjustment. Red points are SNPs above the high significance level (*p *= 0.01 after adjustment). Bright green points are SNPs above the standard significance level (*p *= 0.05 after adjustment). Points below the significance threshold are represented in different colors to denote the different chromosomes.

The GWAS analysis of the hop diversity panel revealed two highly significant molecular markers associated with PM resistance on chromosomes 6 and 8. The SNP on chromosome 6 was positioned at 310,959,501 bp, and two copies of this allele accounted for 36.7% of the variation in the powdery mildew resistance phenotype. The SNP on chromosome 8 was positioned at 70,991,669 bp, and two copies of this allele accounted for 33.0% of the variation in the powdery mildew resistance phenotype. A further molecular marker was associated with PM resistance on chromosome 3 at position 234,277,281 bp, indicating that multiple sources of resistance are present in the diversity panel (Figure 2). The main SNP on chromosome 3 was associated with an effect size of 55.7%.

**FIGURE 3:**
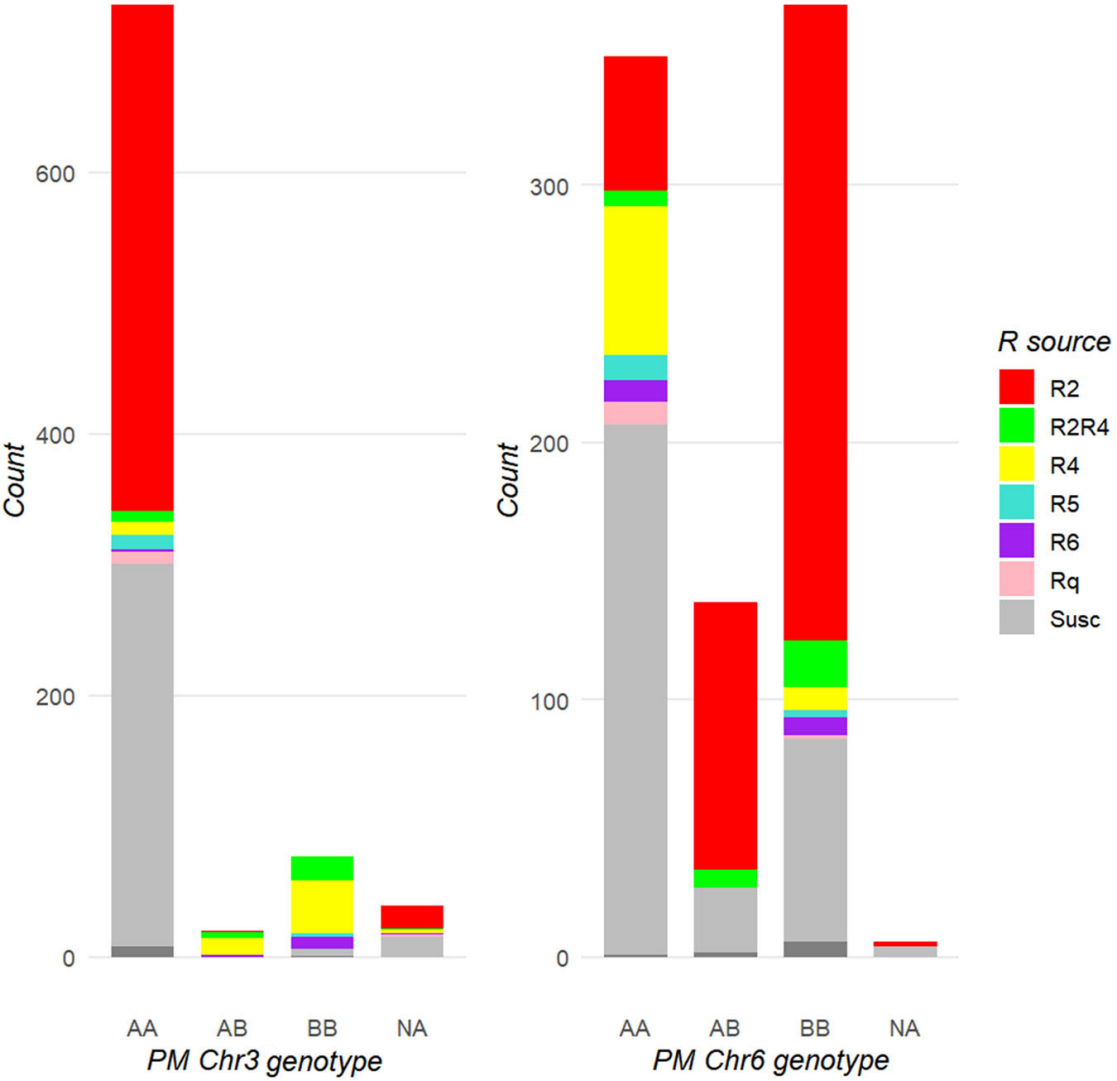
Accession frequency associated with resistance source across the genotypes for the focal powdery mildew resistance markers on chromosomes 3 and 6. AA, homozygote for the susceptible reference allele; AB, heterozygote; BB, homozygote for the alternate resistant allele. Red, R2; green, R2 and R4; yellow, R4; blue, R5; purple, R6; pink, Rq; gray; Susc, susceptible to all powdery mildew races.

The focal SNPs identified in the GWAS were examined for their association with the range of PM race‐specific resistance sources that were represented in the diversity panel (Table 2; Figure 3).

**TABLE 2:** Composition of genome‐wide association study (GWAS) genotype panel individuals based on their powdery mildew resistance source.

PM resistance source	Standard cultivar	No. of individuals	Percentage of individuals
R2	Wye Target	344	46.6
R4	Serebrianka	61	8.3
R2R4	Wye Target/Serebrianka	23	2.4
R5	Early Choice	12	1.6
R6	Nugget	14	1.9
Rq	Cascade	10	1.8
Susceptible	Zenith	272	36.9

*Note*: R2 resistance was confirmed through pathogenicity testing. For other resistance sources, phenotypes were inferred through phylogenetic assessment.

A highly significant association was found between the SNP located on chromosome 6 and the R2 resistance type (*χ* ^2^_(2) _= 276.69, *p* < 0.0001), where individuals carried one or two copies of the resistance allele (AB, BB). The PM‐associated SNP located on chromosome 3 was significantly associated with R4 resistance (*χ* ^2^_(2) _= 489.24, *p* < 0.0001), whereby individuals with one or two copies of the Chr3 marker were closely linked to the R4 resistance type. Furthermore, the Chr3 marker also showed an association with the R6 resistance type (*χ* ^2^_(2)_ = 75.929, *p* < 0.0001). The R6‐source individuals were present at a low frequency in the population (*n* = 14), notwithstanding this R6 resistance still showed an association with the resistance BB and AB genotypes at the chromosome 3 SNP. The PM‐associated marker on chromosome 8 was linked with the R2 resistance (*χ* ^2^_(2)_ = 9.8303, *p* = 0.0078).

**FIGURE 4:**
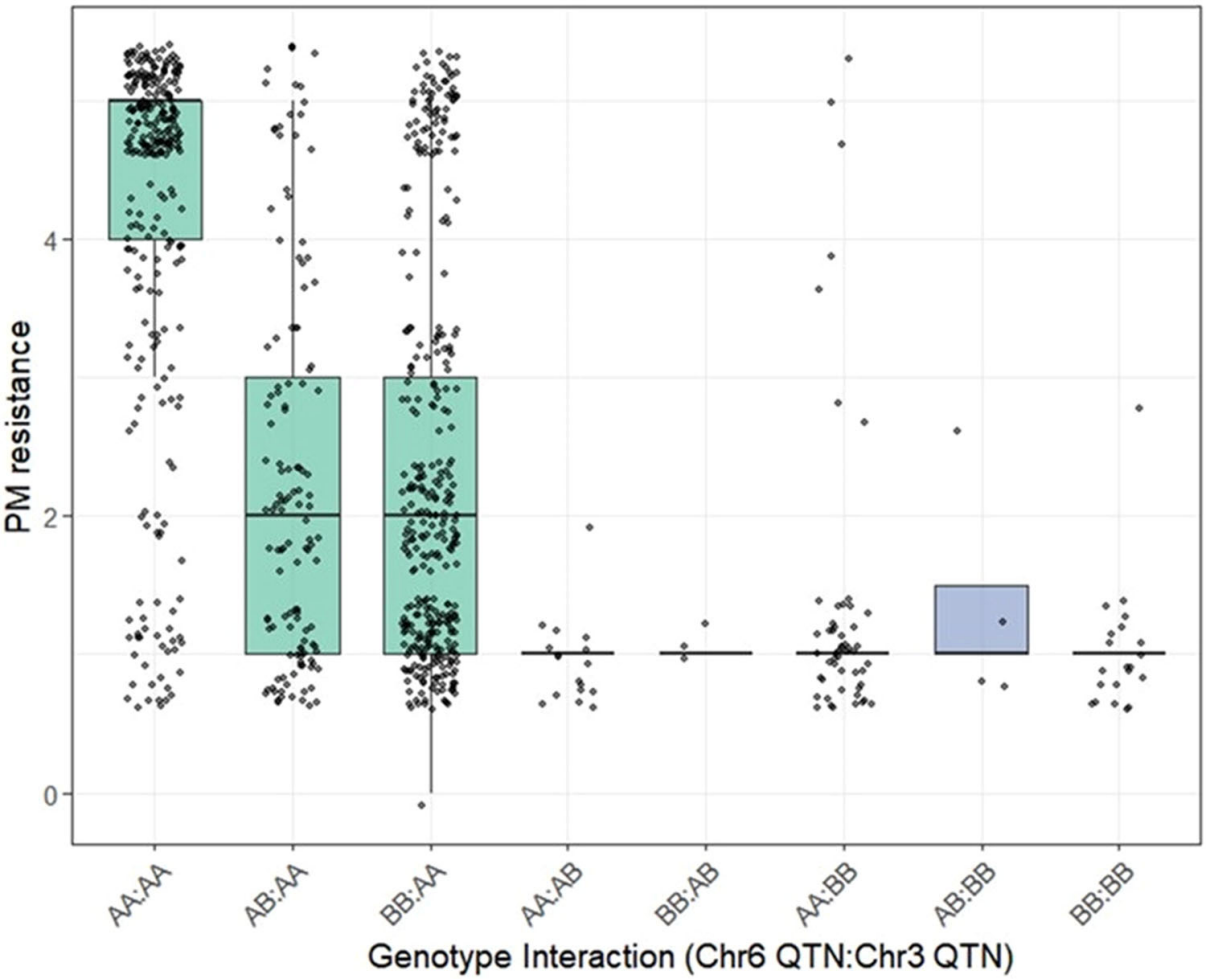
The association of genotype for the R2 associated focal single nucleotide polymorphism (SNP; chromosome 6 at location 310959501 bp) and the R4 associated focal SNP (chromosome 3 at location 23427781 bp) on powdery mildew disease resistance in the genome‐wide association study (GWAS) hop population.

The R2 resistance originally described in Wye Target and the R4 resistance originally described in Serebrianka were demonstrated to represent different disease resistance genes as they are associated with different chromosomes. The R2 type resistance was found to be linked with chromosome 6, whereas the R4 and R6 resistance were both found to be associated with markers on chromosome 3. As illustrated in Figure 4, the R2 locus at chromosome 6 provides partial resistance (AB:AA and BB:AA), which is obfuscated by the presence of the complete resistance provided by R4 at chromosome 3 (**:AB **:BB). No interaction was observed between the resistance focal markers on chromosome 8 and 6, nor 8 and 3 (Table S2).

Putative R genes were identified up to 1 Mb up‐ and downstream from the three powdery mildew linked SNPs. The Cascade reference genome contains a total of 3003 putative disease resistance associated genes (Padgitt‐Cobb et al., 2023) with the largest number of R gene clusters observed near the telomeres of chromosome 8, a region which was identified in this study.

The region covering the window between positions 308.7–312.8 Mb on chromosome 6 contained 426 genes, of which 124 genes had similarity to known UniProt Embryophyta or Pfam genes, and 31 were putative disease resistance genes based on the Biological Processes GO terms associations: “defence response,” “plant type‐hypersensitive response,” and “defence response to fungus, incompatible interaction.” A disease resistance gene cluster was present between the two focal SNPs on chromosome 6 identified via the association mapping of a biparental population and GWAS (Table 3). These three R‐genes were strongly associated with the R2 type resistance and had similarity to the RPM1 resistance gene first characterized in Arabidopsis (Grant et al., 1995). Close to the R2 SNPs were also putative resistance factors such as a glucan endo‐1,3‐beta‐glucosidase protein and a ferredoxin‐3 protein, a KIPK1 protein kinase as well as 108 additional protein‐coding genes. The in‐depth description of the R genes is provided for the highly significant SNP on chromosome 6 found across both the biparent and genome wide association experiments due to the high number of disease resistant genes found in the area (Table 3).

**TABLE 3:** List of defense response associated genes in the region 1 Mb up‐ and downstream from the powdery mildew resistance single nucleotide polymorphism (SNP) on chromosome 6 of the Cascade Dovetail genome.

Cascade chromosome	Start position	End position	Biological processes GO term	Gene name	Pfam domain
6	308774339	308776078	plant‐type hypersensitive response	Disease resistance protein RPM1	NB‐ARC domain
6	308914171	308937907	cell surface receptor signaling pathway	Wall associated receptor kinase (WAK2)	
6	309403556	309404234	defense response	Disease resistance protein RPP2A	NA
6	309408931	309410087	defense response to fungus, incompatible interaction	Disease resistance protein RUN1	Leucine rich repeat
6	309410874	309411327	defense response	Disease resistance protein RRS1	NA
6	309412419	309557962	plant‐type hypersensitive response	TMV resistance protein N	TIR domain
6	309413722	309414455	defense response	Disease resistance protein Roq1	NB‐ARC domain
6	309414731	309417308	defense response to fungus, incompatible interaction	Disease resistance protein RPV1	TIR domain
6	309457900	309459042	defense response to fungus, incompatible interaction	Disease resistance protein RUN1	NB‐ARC domain
6	309551539	309552048	defense response	Disease resistance protein RRS1	Leucine Rich Repeat
6	309625006	309628079	signal transduction	NA	NA
6	**309901108**		**Association mapping SNP**	NA	NA
6	**309901110**		**Association mapping SNP**	NA	NA
6	**309901071**		**Association mapping SNP**	NA	NA
6	309904684	309920514	defense response	Disease resistance protein RGA2	NB‐ARC domain
6	309968819	309969413	plant‐type hypersensitive response	TMV resistance protein N	Leucine Rich repeats (2 copies)
6	309969460	309971484	defense response	Protein VARIATION IN COMPOUND TRIGGERED ROOT growth response	Leucine Rich Repeat
6	309971526	309973694	defense response	Disease resistance protein Roq1	NB‐ARC domain
6	310238296	310244372	defense response	Disease resistance protein RPS2	Leucine Rich repeats (2 copies)
6	310274125	310277256	defense response	Disease resistance protein At4g27190	NB‐ARC domain
6	310278640	310279538	defense response	Disease resistance protein SUMM2	NA
6	310282458	310283600	defense response	Probable disease resistance protein At5g47260	Leucine Rich repeats (2 copies)
6	310857506	310860077	defense response	Probable disease resistance protein At5g47260	NA
6	310862199	310865801	defense response	Disease resistance protein At4g27190	NB‐ARC domain
6	**310959501**		**GWAS focal SNP**	NA	NA
6	**310959493**		**Association mapping SNP**	NA	NA
6	311132367	311135195	plant‐type hypersensitive response	Disease resistance protein RPM1	NB‐ARC domain
6	311204857	311207685	plant‐type hypersensitive response	Disease resistance protein RPM1	NB‐ARC domain
6	311252252	311255080	plant‐type hypersensitive response	Disease resistance protein RPM1	NB‐ARC domain
6	**311711437**		**Association mapping focal SNP**	NA	NA
6	**311711455**		**Association mapping SNP**	NA	NA
6	311751322	311755608	protein phosphorylation	Protein Kinase (KIPK1)	
6	**311767362**		**Association mapping SNP**	NA	NA
6	**311767384**		**Association mapping SNP**	NA	NA
6	311837867	311838476	defense response	Thionin	Plant thionin
6	311859768	311862435	defense response	Patatin‐like protein 3	Patatin‐like phospholipase
6	311873481	311874093	defense response	Thionin	Plant thionin
6	311965538	311966141	defense response	Thionin	Plant thionin
6	312061506	312062297	defense response	Agglutinin‐1	Ribosome inactivating protein
6	312182892	312183521	defense response	Thionin	Plant thionin
6	312271044	312271829	defense response	Ribosome‐inactivating protein gelonin	Ribosome inactivating protein
6	312571945	312580732	defense response	Disease resistance protein At4g27190	NB‐ARC domain

*Note*: The position of the two focal SNPs identified in the association mapping and GWAS population are highlighted with pink shading. Significant powdery mildew resistance DArT SNPs relative to the R genes on chromosome 6 are also marked in this table highlighted with green shading. Gene ID are based upon Cascade gene models (Padgitt‐Cobb et al., 2023).

Abbreviations: GO, Gene Ontology; NA, not applicable.

Chromosome 3 contains a total of 129 putative defense response‐associated genes. The 1‐Mb regions surrounding the R4‐associated SNP on chromosome 3 at 234,277,281 bp contained a total of 50 protein coding genes, of which three had GO term implications of associations with fungal plant defense mechanisms. Chromosome 8 contains 178 defense genes. Of these, there were 59 genes in the 1Mb window near the chromosome 8 SNP located at 70,991,669 bp, of which 23 had GO terms directly associated with fungal plant defense responses. The chromosome 8 focal SNP was positioned near clusters of *serine/threonine‐protein kinase* (*VPS15*), *mitogen‐activated protein kinases* (*M2K6*), and *Glucan endo‐1,3‐beta‐glucosidase* genes. Among the other potential resistance associated genes on chromosome 8 was a gene encoding for an IQD20 protein, which is involved in calcium signaling that modulates defense response.

## 4 DISCUSSION

Our study elucidated the genetic mechanism underlying two race‐specific resistance sources and identified a putative genetic locus for R2 resistance on chromosome 6 using both association mapping and GWAS analysis. We also demonstrated that two genetic loci on chromosome 3 were associated with R4 resistance. These R2 and R4 resistance markers were each associated with single major dominant genes that are associated with resistance across a large and diverse population of hop, and as such, represent valuable genetic markers that can be used in marker‐assisted resistance breeding.

The typical phenotypic expression of resistance to powdery mildew in cultivated hop has been shown to follow a gene‐for‐gene interaction with seven putative R genes previously determined to represent different resistance states (Darby, 2013). The Pilgrim x 316/1/10 progeny used in this study exhibited highly consistent phenotype scores both within and between replicate years, demonstrating that the R2 source of powdery mildew resistance is highly heritable (*H* ^2 ^= 0.99). Moreover, the high heritability of standard cultivars indicates a strong genetic contribution to phenotypic variation among these genotypes. Three recent hop powdery mildew quantitative trait loci (QTL) mapping studies have investigated resistance across populations containing different resistance sources (R1, R4, and R6). Henning et al. (2017, 2011) and Padgitt‐Cobb et al. (2020) reported that there was no significant variation between experimental replicates, suggesting strong genetic control of the trait. Moreover, Havill et al. (2023) reported high heritability of the resistance trait (0.92). In our study, R4 and R6 resistance was associated with the QTL on chromosome 3— it is not clear whether the R4 and R6 resistance sources are caused by the same resistance gene or by two separate, but linked alleles present within the same locus.

Powdery mildew disease scores in the Pilgrim x 316/1/10 family segregated into discrete classes and a chi‐squared test showed a NS difference from a 1:1 segregation ratio between susceptible and resistant phenotypes, which confirmed the action of a single major dominant locus. Notwithstanding this, the variation within the “resistant” phenotypes also indicates the possible presence of additional small effect epistatic disease resistance alleles that interact with the major resistance gene, a finding similar to that previously reported for R4/R6 resistance in hop (Henning et al., 2017).

In the past decade, various attempts have been made to identify the genomic positions of hop powdery mildew R genes via QTL analyses. Henning et al. (2017) utilized genome‐wide SNP markers from a cross between the R4/R6 resistant female Newport and susceptible male counterpart 21110 M and identified three SNPs highly associated with PM resistance and a further 12 SNPs associated with lower impact on PM symptom expression, all of which co‐localized on the same linkage group. It was not clear from the work of Henning et al. (2017) whether the three large effect SNPs correspond to three different R genes or to multiple factors involved in the resistance mechanism of a single resistance source due to the ambiguity of two resistance sources in the cultivar Newport (Wolfenbarger et al., 2014). In more recent QTL mapping attempts, Padgitt‐Cobb et al. (2020) identified SNP markers segregating in the same Newport x 21110 M population while using the more contiguous and complete PacBio Cascade reference genome to map the R4/R6 powdery mildew resistance locus (Padgitt‐Cobb et al., 2023). They identified two linked regions, with the most strongly associated SNPs located on contig 000559F, which corresponds to the 17‐ to 18‐Mb region of chromosome 3 in the current Dovetail genome, downstream of several pathogenesis‐related genes.

In a QTL mapping study conducted by Havill et al. (2023), a single QTL was also identified on chromosome 3 of the Cascade genome using a biparental population developed between the R1 resistant female Zenith and the susceptible male breeding line USDA 21058 M. The QTL showed a strong association with R1‐mediated resistance. This R1 genetic locus was observed to be a discrete locus to that associated with the R4/R6 resistance source. As these R loci were identified on different chromosomes, we can be confident that the R4/R6 and R1 resistance loci represent a discrete resistance source to the R2 examined here.

Notably, other researchers studying an undefined resistance source in the cultivar Comet have identified a region of chromosome 6 similar to the one identified in this study in Pilgrim (Henning et al., 2024). Their focal SNP was positioned at 313,335,048 bp, which is 2.4 Mb away from our focal SNP. In contrast to the study presented here, their region was not linked to the R2 resistance source. The Comet resistance source exhibited polygenic resistance with additional QTL found on multiple chromosomes with the strongest QTL associated with the 6‐Mb region between 308 and 314 Mb on chromosome 6. By contrast, the resistance locus identified in Pilgrim was on chromosome 6 and could be narrowed to a region between 309 and 313 Mb with a shifted focal SNP that sheds greater light on potential causative resistance genes. Of particular note, while both Pilgrim and Comet confer durable gradient resistances to powdery mildew infection, the two genotypes exhibit resistance against different *P. macularis* race types, illustrating the potential that the two resistance sources may operate via distinct resistance mechanisms.

The resistance in Pilgrim can be traced back to the accession OB79, within which the R2 resistance was first identified (Darby, 1996; Keyworth et al., 1953). This R2 source has been introgressed into a line with superior qualities through six generations of continuous R2‐resistant × high‐quality but susceptible crosses, including the 11/65 cross, which led to Wye Target. Both Wye Target and Pilgrim contain the R2‐type resistance derived from OB79, and the segregation of resistance follows a dominant major gene pattern in both genotypes. However, Wye Target exhibits complete immunity when inoculated under glasshouse conditions with the powdery mildew isolate derived from Zenith with virulence pattern V1, V3, and Vb. By contrast, Pilgrim displays poor‐to‐very poor resistance to this isolate, suggesting a more qualitative resistance in Wye Target and a partial or quantitative resistance in Pilgrim (Figure S1). We observed a cluster of R genes in the R2 region. It is possible that Wye Target carries an additional mutation that leads to higher expression or increased efficacy of the resistance gene at the R2 locus, leading to stronger resistance.

The R2 locus spans a 3.4‐Mb interval, within which we have identified a cluster of three *RPM1* genes, each representing a promising resistance gene candidate (Debener et al., 1991; Grant et al., 1995). RPM1 proteins operate through a highly potent resistance mechanism that is reminiscent of the large effect disease response observed in R2 resistant hops. RPM1 works through detecting specific pathogen effectors which in this case act as determinants of avirulence. Detection leads to a calcium influx, oxidative burst, and the hypersensitive response (HR) within 5 h of infection. This HR results in localized plant cell collapse, thus preventing the spread of the pathogen (Grant et al., 2000).

*RPM1* genes have been extensively studied as model disease resistance genes that were first found to play a role in protecting *Arabidopsis thaliana* from *Pseudomonas syringae* bacterial infection (Grant et al., 1995). Since they were first identified, RPM1 homologues have also been found to be conserved in different plant species (Dangl et al., 1992). Notably, *RPM1* homologues in wheat have been implicated in resistance to powdery mildew disease caused by the fungal biotrophic pathogen *Blumeria graminis* f. sp. *tritici*; in particular, TaRPM1 is induced strongly by powdery mildew infection (Nie & Ji, 2019; Simeone et al., 2020). Given the role of *RPM1* genes in providing complete resistance to powdery mildew in wheat and the commensurate HR resistance response that we observe, our three target genes represent strong candidates for endowing R2 resistance to *P. macularis* in hop. Indeed, localized cell necrosis is a symptom often observed within hop cultivars carrying R2 and Rsov resistances to powdery mildew (Darby, 2013).

RPM1 forms part of a protein complex that is tethered to the cytosolic side of the plasma membrane, and the protein itself contains coiled‐coil, nucleotide binding site and leucine rich repeat domains (CC‐NB‐LRR), which are well characterized as recognizing pathogen effector proteins and inducing HR (J. D. G. Jones & Dangl, 2006; Gao et al., 2011). RPM1 guards the protein RIN4 specifically; effectors cause ADP‐ribosylation of RIN4, leading to phosphorylation of the Thr‐166 residue of RIN4, which in turn leads to RPM1 conformational change and the localized cell death response, which regulates plant immunity (Ray et al., 2019; Redditt et al., 2019). It has been suggested that RPM1 provides an effective but sometimes overly sensitive disease response (McDowel & Simon, 2006); unlike other resistance genes, the maintenance of RPM1 has been shown to have a fitness cost in *A. thaliana* (Tian et al., 2003). It is not clear whether this fitness cost is associated with the R2 hop powdery mildew resistance; however, if present, it may be possible to introduce additional genes that mitigate any fitness cost.

A cluster of seven R genes surrounded by seven putative *glucan endo‐1,3‐beta‐glucosidase* genes has been reported on chromosome 3 by Padgitt Cobb et al. (2020) in a QTL investigation of the R6 resistance. In this case, the nearest genes to the powdery mildew HLPM_10681892 marker were three *Accelerated Cell Death 6* (*ACD6*)‐like protein‐coding genes. In a similar fashion to RPM1, ACD6‐like proteins function as cell death regulators, which is an important host defense mechanism in Arabidopsis against pathogens. Programmed cell death (necrotic spots) is a type of plant HR induced by R protein‐mediated systemic acquired resistance (Liu et al., 2010).

As mentioned previously, hop powdery mildew has been observed to represent a defined race structure with *P. macularis* race types corresponding to the seven described resistance sources (R1–R6 and Rb [blister]; Darby, 2001; Gent et al., 2017). Typically, single gene resistance is prone to break down within the field. To combat this, the stacking of resistance genes can lead to more durable resistance (Dangl & Jones, 2001). We have identified SNPs associated with R2 and R4 resistance sources across the United Kingdom diversity panel. This information will enable the development of molecular markers tagging resistance for the creation of robust powdery mildew resistant hop cultivars containing multiple resistance genes. There is a differential distribution of *P. macularis* race types across the globe, leading breeders to seek specific resistance genes that are effective against the dominant races of powdery mildew (Gent et al., 2019). In particular, the R2 resistance gene is likely to be highly effective against Pacific Northwest US *P. macularis* populations where the R1 and R2 resistances remain effective (Havill et al., 2023; Wolfenbarger et al., 2016).

We have identified the same genetic component controlling R2 powdery mildew resistance on chromosome 6 in two different populations. We identified three candidate disease resistance genes with RPM1 annotations neighboring the R2 focal SNP that are strong candidates for downstream investigation. Further to this, we identified a molecular region that was significantly linked to the R4 powdery mildew resistance phenotype. The molecular region identified in the GWAS has been identified across a diverse set of 736 individuals and so can be considered to be “validated” as they illustrate a strong association between the markers and the phenotype across a wide diversity of germplasm. When taken together, the results reported here can be used to implement marker‐assisted breeding in order to generate powdery mildew resistant hop cultivars.

## AUTHOR CONTRIBUTIONS

**Klara Hajdu**: Conceptualization; data curation; formal analysis; investigation; writing—original draft; writing—review and editing. **John Connell**: Formal analysis. **Peter Darby**: Investigation; resources; supervision. **Michael Baldock**: Data curation. **Andrew Armitage**: Supervision. **Alastair Ainslie**: Formal analysis. **Sarah Blackburn**: Data curation. **Carol Wagstaff**: Supervision. **Helen Cockerton**: Conceptualization; project administration; supervision; writing—review and editing.

## CONFLICT OF INTEREST STATEMENT

The authors declare no conflicts of interest.

## Supporting information



Supplementary Figure 1. Hop powdery mildew disease severity scores recorded on the PM differential standard cultivars and parents for the mapping population. Numbers represent disease scores. Top row 5‐ “Susceptible” genotypes with no resistance background 316/1/10, ‘Northern brewer’ and with R1 ‘Zenith’ and R3 type ‘Challenger’ resistances showing necrosis, chlorosis, merging lesions, sporulation on petioles and under the leaves. Middle row: 2‐ “Poor resistant” cultivar with weak R2 resistance ‘Pilgrim’ showing slight chlorosis, 3‐ “Very poor resistant” cultivar with weak R2 and Rsov resistances ‘Sovereign’ showing chlorosis, blistering and aborted sporulation. 4‐ “Query susceptible” cultivar with Rq ‘Cascade’ type resistance showing ghost sporulation in glasshouse condition, weak resistance in field conditions. Bottom row:1‐ “Resistant” cultivars with R5 resistance ‘Early Choice’, R6 ‘Nugget’, R4 ‘Serebrianka’ and strong R2 type ‘Wye Target’ resistance showing no infection.
Supplementary Figure 2. Phenotypic BLUEs calculated for 12 powdery mildew standard cultivars expressing differential responses to the *P. macularis* ‘Zenith’ isolate, included in multiple GWAS experimental years, with the R2 standard ‘Wye Target’, the R1 standard ‘Zenith’, and universal susceptible cultivar ‘Northern Brewer’ included in all experimental years. Additional standards were: R4 standard ‘Serebrianka’, R5 standard ‘Early Choice’ and R6 standard ‘Nugget’, R3 standard ‘Challenger’, susceptible ‘Yeoman’, R2 resistant ‘Pilgrim’, R2Rsov resistant ‘Sovereign’, Rq ‘Cascade’ and susceptible ‘Columbus’.
Supplementary table 1. Frequency of Use for Reference Cultivars in Powdery Mildew Screening Experiments between 1982–2022
Supplementary table 2. Statistical significance of pairwise interactions among the four powdery mildew resistance markers identified on chromosomes 6, 3, and 8.

Supplementary Material

Supplementary Material

Supplementary Material

Supplementary Material

## Data Availability

The data are available in Supporting Information files. Bioinformatics pipelines for genotyping are available at the GitHub repository (https://github.com/jcniabemr/IUKHop).
