# Supplementary material for: Genetic components associated with R2 and R4 powdery mildew resistance in hop
Source: Plant Genome. 2026 Feb 11;19(1):e70180. doi: 10.1002/tpg2.70180 (PMC12895981; doi:10.1002/tpg2.70180)
Supplement: Supplementary file 1 [file TPG2-19-e70180-s001.docx]

**SUPPLEMENTAL MATERIAL**

Supplementary Figure 1. Hop powdery mildew disease severity scores recorded on the PM differential standard cultivars and parents for the mapping population. Numbers represent disease scores. Top row 5- “Susceptible” genotypes with no resistance background 316/1/10, ‘Northern brewer’ and with R1 ‘Zenith’ and R3 type ‘Challenger’ resistances showing necrosis, chlorosis, merging lesions, sporulation on petioles and under the leaves. Middle row: 2- “Poor resistant” cultivar with weak R2 resistance ‘Pilgrim’ showing slight chlorosis, 3- “Very poor resistant” cultivar with weak R2 and Rsov resistances ‘Sovereign’ showing chlorosis, blistering and aborted sporulation. 4- “Query susceptible” cultivar with Rq ‘Cascade’ type resistance showing ghost sporulation in glasshouse condition, weak resistance in field conditions. Bottom row:1- “Resistant” cultivars with R5 resistance ‘Early Choice’, R6 ‘Nugget’, R4 ‘Serebrianka’ and strong R2 type ‘Wye Target’ resistance showing no infection.

Supplementary Figure 2. Phenotypic BLUEs calculated for 12 powdery mildew standard cultivars expressing differential responses to the *P. macularis* ‘Zenith’ isolate, included in multiple GWAS experimental years, with the R2 standard ‘Wye Target’, the R1 standard ‘Zenith’, and universal susceptible cultivar ‘Northern Brewer’ included in all experimental years. Additional standards were: R4 standard ‘Serebrianka’, R5 standard ‘Early Choice’ and R6 standard ‘Nugget’, R3 standard ‘Challenger’, susceptible ‘Yeoman’, R2 resistant ‘Pilgrim’, R2Rsov resistant ‘Sovereign’, Rq ‘Cascade’ and susceptible ‘Columbus’.

Supplementary table 1. Frequency of Use for Reference Cultivars in Powdery Mildew Screening Experiments between 1982–2022

| **PM Standard Genotype** | **PM resistance source** | **Frequency (No. of years tested)** |
| --- | --- | --- |
| **Northern Brewer** | Susceptible | 38 |
| **Wye Target** | R2 | 38 |
| **Zenith** | R1 | 38 |
| **Challenger** | R3 | 28 |
| **Early Choice** | R5 | 33 |
| **Serebrianka** | R4 | 32 |
| **Nugget** | R6 | 31 |
| **Yeoman** | Susceptible | 23 |
| **Pilgrim** | R2 | 17 |
| **Sovereign** | R2 Rsov | 11 |
| **Cascade** | Rq | 8 |
| **Columbus** | Susceptible | 7 |

Supplementary table 2. Statistical significance of pairwise interactions among the four powdery mildew resistance markers identified on chromosomes 6, 3, and 8.
